# Bexarotene Impairs Cognition and Produces Hypothyroidism in a Mouse Model of Down Syndrome and Alzheimer’s Disease

**DOI:** 10.3389/fphar.2021.613211

**Published:** 2021-04-15

**Authors:** Verónica Vidal, Alba Puente, Susana García-Cerro, María Teresa García Unzueta, Noemí Rueda, Javier Riancho, Carmen Martínez-Cué

**Affiliations:** ^1^ Department of Physiology and Pharmacology, Faculty of Medicine, University of Cantabria, Santander, Spain; ^2^ CIBERSAM, Madrid, Spain; ^3^ Department of Clinical Biochemistry, University Hospital Marques de Valdecilla-IDIVAL, Santander, Spain; ^4^ Neurology Service, Hospital Sierrallana-IDIVAL, Torrelavega, Spain; ^5^ Department of Medicine and Psychiatry, University of Cantabria, Santander, Spain; ^6^ CIBERNED, Madrid, Spain

**Keywords:** Down syndrome, Alzheheimer’s disease, bexarotene, cognition, hypothyroidism

## Abstract

All individuals with Down syndrome (DS) eventually develop Alzheimer’s disease (AD) neuropathology, including neurodegeneration, increases in *β*-amyloid (Aβ) expression, and aggregation and neurofibrillary tangles, between the third and fourth decade of their lives. There is currently no effective treatment to prevent AD neuropathology and the associated cognitive degeneration in DS patients. Due to evidence that the accumulation of Aβ aggregates in the brain produces the neurodegenerative cascade characteristic of AD, many strategies which promote the clearance of Aβ peptides have been assessed as potential therapeutics for this disease. Bexarotene, a member of a subclass of retinoids that selectively activates retinoid receptors, modulates several pathways essential for cognitive performance and Aβ clearance. Consequently, bexarotene might be a good candidate to treat AD-associated neuropathology. However, the effects of bexarotene treatment in AD remain controversial. In the present study, we aimed to elucidate whether chronic bexarotene treatment administered to the most commonly used murine model of DS, the Ts65Dn (TS) mouse could reduce Aβ expression in their brains and improve their cognitive abilities. Chronic administration of bexarotene to aged TS mice and their CO littermates for 9 weeks diminished the reference, working, and spatial learning and memory of TS mice, and the spatial memory of CO mice in the Morris water maze. This treatment also produced marked hypoactivity in the plus maze, open field, and hole board tests in TS mice, and in the open field and hole board tests in CO mice. Administration of bexarotene reduced the expression of Aβ1-40, but not of Aβ1-42, in the hippocampi of TS mice. Finally, bexarotene increased Thyroid-stimulating hormone levels in TS mice and reduced Thyroid-stimulating hormone levels in CO mice, while animals of both karyotypes displayed reduced thyroxine levels after bexarotene administration. The bexarotene-induced hypothyroidism could be responsible for the hypoactivity of TS and CO mice and their diminished performance in the Morris water maze. Together, these results do not provide support for the use of bexarotene as a potential treatment of AD neuropathology in the DS population.

## Introduction

Down syndrome (DS), the most common genetic cause of intellectual disability, is caused by a partial or complete triplication of the human chromosome 21 (Antonarakis et al., 2020). The cognitive alterations found in DS are primarily caused by prenatal changes in central nervous system growth and differentiation (Contestabile et al., 2007; Guidi et al., 2008; Haydar and Reeves, 2012; Lott, 2012; Rueda et al., 2012; Hibaoui et al., 2014; Stagni et al., 2018). Furthermore, these alterations are aggravated in later life stages. By the fourth decade of their lives, all individuals with DS develop Alzheimer’s disease (AD) neuropathology, including the accumulation of amyloid plaques comprising *β*-amyloid (Aβ) peptides, neurofibrillary tangles (NFTs) formed by insoluble deposits of abnormally hyperphosphorylated tau, synaptic and neuronal loss, reduced neurogenesis, regional atrophy, and chronic microglial-driven inflammatory response, which leads to dementia (Teipel and Hampel, 2006; Sabbagh et al., 2011; Cenini et al., 2012; Lott, 2012; Wilcock and Griffin, 2013; Moreno-Jiménez et al., 2019).

There is currently no effective treatment for AD neuropathology and/or to delay the associated cognitive impairment in demented patients with or without DS. There is, therefore, a pressing need to search for new strategies to prevent or delay the course of this disease. In this context, DS emerges as a valuable optimal model to study AD pathology. Importantly, the knowledge obtained in animal models of DS will help to foster a better understanding of the causes of neurodegeneration and dementia, both in DS and in sporadic AD, and assist in the development of new therapeutic strategies to treat them.

Several mouse models of DS have been developed (Antonarakis et al., 2020). However, none of them completely reproduces DS pathophysiology, probably due to the incomplete synteny between Hsa21 and homologous mouse regions (Gupta et al., 2016; Antonarakis et al., 2020). Among these mouse models, the one that has been most characterized and which best reproduces DS cognitive and neurobiological phenotypes is the partially trisomic Ts65Dn (TS) mouse (Sturgeon and Gardiner, 2011; Rueda et al., 2012; Gupta et al., 2016). Consequently, this mouse model is the most commonly used in preclinical studies to test the ability of different pharmacotherapies to rescue these phenotypes (Sturgeon and Gardiner 2011; Gupta et al., 2016); which allows a comparison of the relative efficacy of the different therapeutic strategies in similar circumstances. Among the most relevant DS phenotypes that TS mice replicate are alterations in behavior, learning, and memory, brain morphology and hypocellularity, neurogenesis, neuronal connectivity, and electrophysiological and neurochemical processes (Bartesaghi et al., 2011; Rueda et al., 2012). As in DS, many of these phenotypes appear at prenatal stages, and during adulthood, the TS mouse also exhibits increased levels of APP, Aβ peptides, tau hyperphosphorylation, and neurodegeneration (Lockrow et al., 2009; Shichiri et al., 2011; Corrales et al., 2013, 2014; García-Cerro et al., 2017). However, these animals do not present amyloid plaques or NFTs, as occurs in DS (Seo and Isacson, 2005; Netzer et al., 2010; Millan Sanchez et al., 2012; Rueda et al., 2012). TS mice also exhibit increased neuroinflammation due to microglial activation that alters the expression of inflammatory cytokines in the brain (Hunter et al., 2004; Lockrow et al., 2011; Roberson et al., 2012; Rueda et al., 2018).

Several pharmacotherapies have been reported to rescue the neuromorphological and cognitive deficits in murine models of DS (Malberg et al., 2000; Clark et al., 2006; Bianchi et al., 2010a, 2010b; Contestabile et al., 2013; Martínez-Cué et al., 2013; Guidi et al., 2014; Stagni et al., 2015, 2016, 2017, 2019; Navarro-Romero et al., 2019; Zhou et al., 2019). However, some of these drugs did not demonstrate any clinical benefit when tested in humans, or cannot be safely administered to individuals with DS (Gardiner, 2014; Tayebati et al., 2019).

Based on the hypothesis that the accumulation of Aβ aggregates in the brain might trigger the AD neurodegenerative cascade, many strategies that promote the clearance of Aβ peptides have been assessed as potential therapeutics for this disease (Golde et al., 2010). In AD brains, one of the causes of the accumulation of Aβ aggregates is their defective clearance from the brain, a process normally facilitated by apolipoprotein E (ApoE). Indeed, the major genetic risk factor for sporadic AD is a polymorphism of ApoE (Bu, 2009; Liu et al., 2013). ApoE contributes to the maintenance of brain homeostasis through numerous pathways, including the regulation of cholesterol, glucose metabolism, synaptic plasticity, neurogenesis, inflammatory responses, and Aβ metabolism (Bu, 2009; Huang and Mucke, 2012; Liu et al., 2013). In humans, there are three ApoE isoforms: E2, E3, and E4. The different ApoE isoforms induce different effects on Aβ clearance and cytoskeleton stability (Mahley et al., 2006). In the AD population, the presence of the ApoE4 isoform correlates with a higher probability of developing dementia and an earlier onset of cognitive decline (Bu, 2009). Furthermore, healthy ApoE4 carriers display reduced cognitive function during aging when compared to individuals carrying other ApoE isoforms (Zehnder et al., 2009; Reinvang et al., 2010).

The expression of ApoE is regulated by the ligand-activated nuclear receptors Peroxisome Proliferator-Activated Receptor-c (PPARγ), the Liver X Receptor (LXR), and the Retinoid X Receptor (RXR) (Liang et al., 2004; Zhao et al., 2014). These receptors regulate ApoE expression by forming heterodimers with each other or with other nuclear receptors such as Retinoid Acid Receptor (RAR), Thyroid Hormone Receptor (TR), or Vitamin D Receptor (VDR) (Chawla et al., 2001; Szanto et al., 2001; Sussman and de Lera, 2005; Evans and Mangelsdorf, 2014).

It has been suggested that agonism of PPARγ, LXR, and RXR could promote the clearance of Aβ (Koldamova and Lefterov, 2007). In mouse models of AD, LXR and RXR agonists ameliorate memory deficits and decrease the Aβ load due to the up-regulation of ApoE (Koldamova et al., 2005; Jiang et al., 2008; Fitz et al., 2010; Cramer et al., 2012), and the two PPARγ agonists used for the treatment of diabetes, Pioglitazone and Rosiglitazone, reduce Aβ levels and improve cognitive functions in AD mice (Mandrekar-Colucci and Landreth, 2011). Thus, drugs modulating the activity of these receptors and the expression of ApoE appear to be promising strategies in the treatment of AD.

The activation of RARs and RXRs receptors by Vitamin A (retinol) and its derivatives (retinoids) regulates the expression of different genes involved in essential cellular processes, such as chromatin remodeling, protein metabolism, intracellular signaling, synaptic homeostasis, and inflammation (Lane and Bailey, 2005). In AD, retinoid signaling is altered (Sodhi and Singh, 2014), which could be responsible for the disruption in protein metabolism, synaptic alterations, and activated astroglia, which are hallmarks of this disease. This data suggests the potential efficacy of retinoids to reduce AD neuropathology by modulating Aβ, reducing neuroinflammation, and preventing synaptic and neurotransmitter alterations.

Bexarotene is a member of a subclass of retinoids that selectively activates RXRs. Bexarotene selectively binds and activates retinoid X receptor subtypes (RXRα, RXRβ, RXRγ). As previously mentioned, these receptors can form heterodimers with other nuclear receptors, which after being activated could function as transcription factors that regulate the expression of genes that control multiple cellular functions which are altered in AD (de Urquiza et al., 2000; Chai et al., 2008).

Bexarotene is approved by the FDA for the treatment of cutaneous lymphoma (Henney, 2000; Farol and Hymes, 2004), and has beneficial effects in several models of neurodegenerative diseases including Parkinson’s disease, Huntington’s disease, Amyotrophic Lateral Sclerosis (ALS), ischemic and hemorrhagic stroke, and epilepsy (McFarland et al., 2013; Bomben et al., 2014; Certo et al., 2015; Riancho et al., 2015; Dickey et al., 2017; Liu et al., 2019; Martín-Maestro et al., 2019; Zuo et al., 2019; Chang et al., 2020; Muñoz-Cabrera et al., 2020).

Bexarotene modulates several pathways essential for cognitive performance, inflammatory response, and Aβ clearance (Lefterov et al., 2015; Riancho et al., 2016). However, the effects of bexarotene in AD are discordant. The first study of the effects of bexarotene in a mouse model of AD reported a reduction in the number of Aβ plaques, in the levels of soluble Aβ, and facilitation of Aβ clearance, through the ApoE related mechanism. Significantly, these changes were accompanied by cognitive improvement (Cramer et al., 2012). However, subsequent investigations failed to completely reproduce these findings (Fitz et al., 2013; Landreth et al., 2013; Price et al., 2013; Tesseur et al., 2013; Veeraraghavalu et al., 2013). None of the subsequent studies was able to replicate the bexarotene-induced reduction of Aβ plaques (Fitz et al., 2013; Landreth et al., 2013; Price et al., 2013; Tesseur et al., 2013; Veeraraghavalu et al., 2013). However, some studies demonstrated the facilitation of the clearance of soluble Aβ peptides by ApoE, the reversal of cognitive deficits (Cramer et al., 2012; Fitz et al., 2013; Muñoz-Cabrera et al., 2020), and the prevention of the effect of ApoE4 on tau hyperphosphorylation (Boehm-Cagan and Michaelson, 2014) after bexarotene administration to mouse models of AD. Recently, it has been reported that OAB-14, a bexarotene derivative, reduces cognitive impairments by increasing *β*-amyloid clearance in APP/PS1 mice (Yuan et al., 2019). Finally, regarding the use of bexarotene in patients with cognitive impairment, two clinical trials in healthy volunteers and patients with mild to moderate AD were conducted (NCT01782742 and NCT02061878), reporting that this retinoid agonist exerted cognitive benefits in these patients (Cummings et al., 2016; Pierrot et al., 2016).

Thus, although bexarotene presents as a very interesting potential drug against AD, its role and efficacy in the treatment of AD remain unclear. In this study, because DS is a useful model for investigating sporadic AD and potential pharmacotherapies to treat this disorder, we aimed to evaluate the effects of chronic bexarotene administration to aged TS mice, which display many hallmarks of AD, on their cognitive and behavioral abilities and their brain Aβ loads. Because the effects of this drug on the cognitive performance of these mice after bexarotene administration were the opposite of the expected effects, we hypothesized that this result might have been due to the induction of hypothyroidism, and we therefore evaluated thyroid hormones levels in TS and CO mice.

## Methods

### Animals and Treatments

This study was approved by the Cantabria University Institutional Laboratory Animal Care and Use Committee and performed in accordance with the Declaration of Helsinki and the European Communities Council Directive (86/609/EEC).

TS mice were generated and karyotyped as previously described in Rueda et al. (2018).

A total of 39 TS and CO mice were assigned to one of four experimental groups: TS-Bexarotene (TS Bx, *n* = 8), TS-vehicle (TS Vh, *n* = 12), CO-Bexarotene (CO Bx, *n* = 11), and CO-vehicle (CO Vh, *n* = 8).

When the mice were 10 months old, the pharmacological treatments were initiated. For nine weeks, TS and CO mice were subcutaneously treated daily with 100 mg/kg of bexarotene (Targretin®, Eisai Inc. Woodcliff Lake, NJ, United States), or the same volume of vehicle (saline). After completing the behavioral studies, the animals were euthanized by cervical dislocation. The brains of seven animals from each group were used for the analyses of Aβ1-40 and Aβ1-42 levels, and serum samples from five animals per group were used to determine Thyroid-stimulating hormone and thyroxine levels.

### Behavioral Analyses

All the behavioral analyses were performed 1 h after the administration of the treatments.

#### Spatial Learning and Memory: Morris Water Maze

**FIGURE 1:**
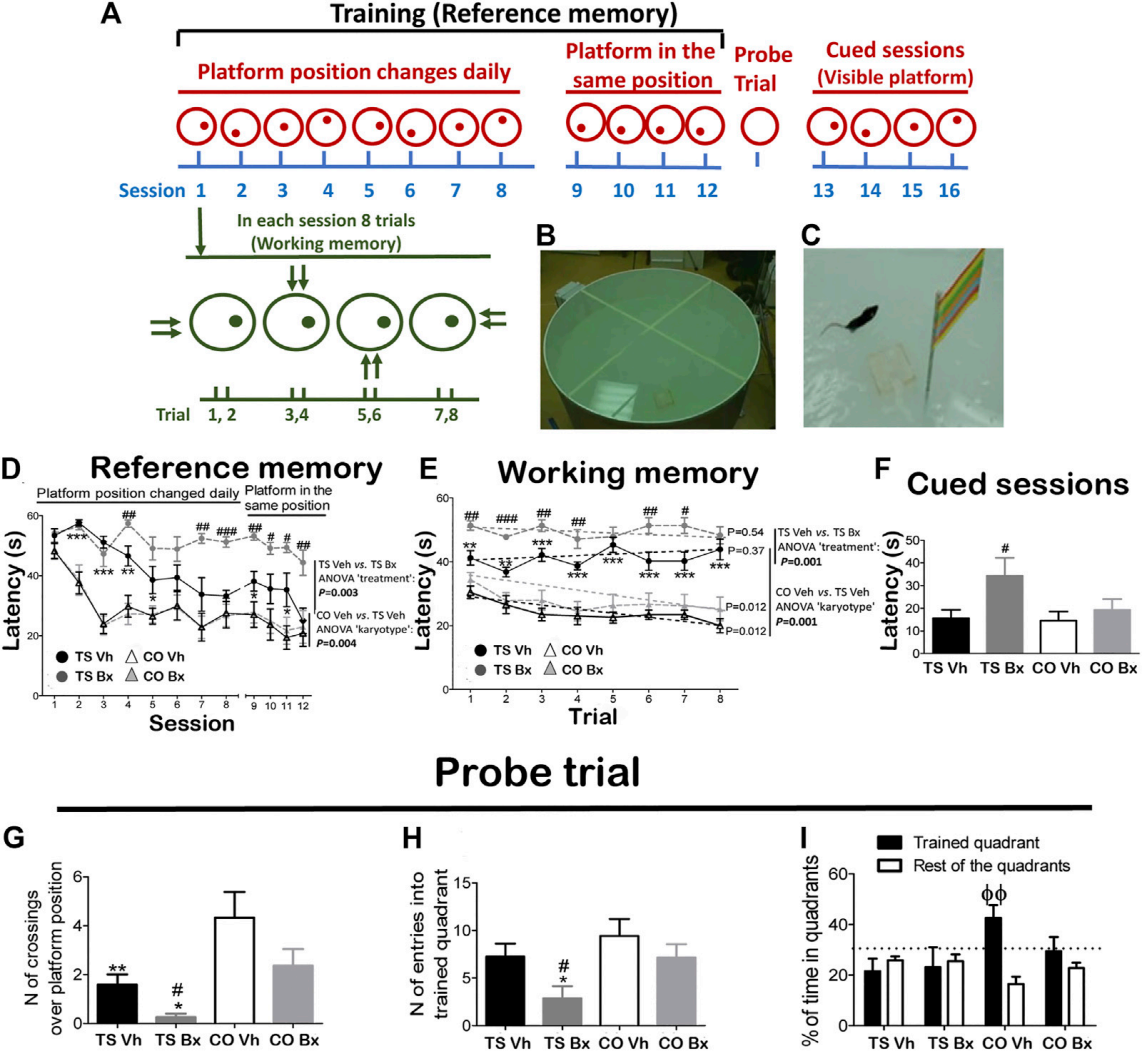
Schematic drawing of the MWM protocol **(A)**, and representative image of the apparatus during the training **(B)**, and cued **(C)** sessions. Mean ± S.E.M of the latency to reach the platform during the twelve acquisition sessions **(D)**, during each trial of the first eight acquisition sessions **(E)**, and mean latencies to reach the platform during the four cued sessions **(F)**, of the number of crossings over the platform position **(G)**, the number of entries in the trained quadrant **(H)**, and the mean percentage of time spent in the trained quadrant vs. the mean time spent in the rest of the quadrants **(I)**, during the probe trial by TS and CO mice under bexarotene or vehicle treatment. *: *p* < 0.05, **: *p* < 0.01, ***: *p* < 0.001: TS Vh vs. CO Vh or TS Bx vs. CO Bx; #: *p* < 0.05, ##: *p* < 0.01, ###: *p* < 0.001 TS Bx vs. TS Vh; ϕϕ: *p* < 0.01 trained quadrant vs. rest of the quadrants. Fisher’s LSD post-hoc tests. On the right side of the A and B figures, the *p*-value of the difference between the TS Bx and the TS Vh, and between the TS Vh and CO Vh learning curves across the twelve sessions (RM ANOVAs) is shown. In the B figure, the dotted lines and the *p*-values beside them represent the significance of the change in latency across the trials (RM ANOVA ‘trial’ of each learning curve). The dotted lines in figure C represent the chance level, i.e. a probability equal to 25%.

To evaluate spatial learning and memory, a modified version of the Morris water maze (MWM) using the same apparatus, experimental conditions, and protocol described by Martínez-Cué et al. (2013) was used (Figures 1A–C).

The animals were submitted to sixteen consecutive daily sessions: 12 acquisition sessions (platform submerged in a different position in each session), four acquisition sessions (platform submerged in the same position), a probe trial, and four cued sessions (platform visible). This protocol allows discrimination between reference memory (between-session performance), working memory (within-session performance), and spatial memory (probe trial). The *Anymaze* computerized tracking system (Stoelting, Wood Dale, IL, United States) was used to analyze the mouse trajectories and to measure the escape latency for each animal in each trial.

#### Fear Conditioning Test

**FIGURE 2:**
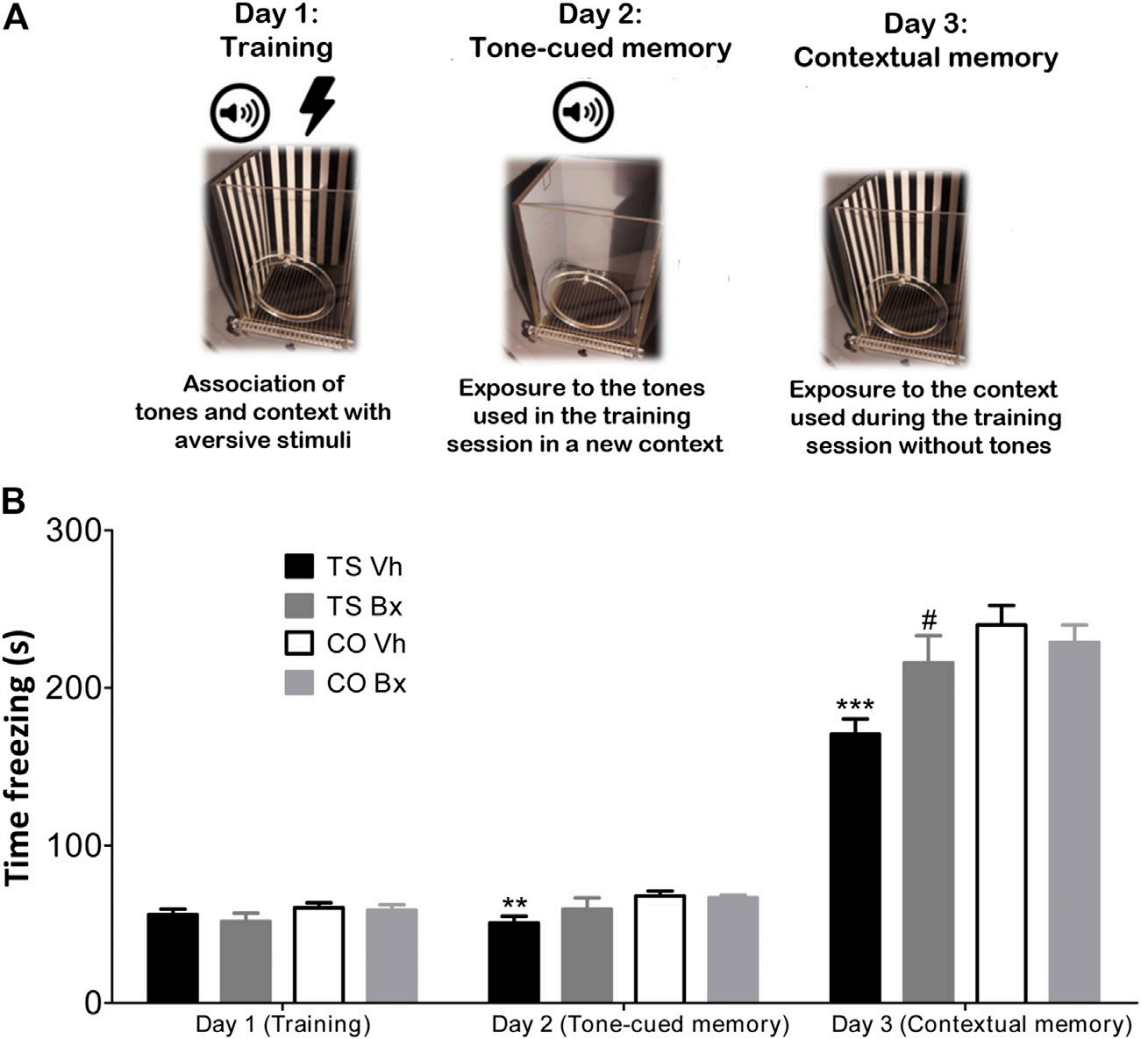
Schematic protocol of the Fear Conditioning Test **(A)**. Mean ± S.E.M of the time that TS and CO mice under bexarotene or vehicle treatment spent freezing during the training session, and in the tone-cued and the contextual memory tests sessions in the Fear Conditioning test **(B)**. **: *p* < 0.01; ***: *p* < 0.001 TS Vh vs. CO Vh, #: *p* < 0.05 TS Bx vs. TS Vh; Fisher’s LSD post-hoc tests.

For the Fear Conditioning experiment, the procedure was performed as described by Vidal et al. (2018) (Figure 2A). Contextual and tone-cued fear conditioning tests were performed using the Fear Conditioning apparatus (Stoelting) and the *AnyMaze* Video Tracking System. The mice underwent three days of testing: a training day, a tone-cued-in-a-novel-context testing day, and a contextual testing day. In the training session, each mouse received five tone-shock pairings. The shock (0.5 mA, 50 Hz, 2 s) was delivered 18 s after the end of the tone (70 dB, 2 kHz, 20 s). In the following session (the tone-cued testing day), each mouse was placed in a novel context for 3 min and they were exposed to three tones identical to the ones of the training day, but they did not receive any shock. In the last session, each mouse was placed in a context identical to the one used in the training day for 5 min, but they were not exposed to any tone or shock. The time that the animals spent freezing in the testing sessions was used as a measure of the memory of the association between the tones and shocks, and the tone and the environment, respectively.

#### Anxiety and Motor Activity

##### Plus Maze

To analyze anxiety and motor activity, the same apparatus and protocol described in Martínez-Cué et al. (2013) was used. During a single 5-min trial, the initial freezing time, the distance traveled, and the time spent in the open and closed arms, and the number of risk assessment behaviors (Stretch-Attend Postures (SAPs) and Head Dippings (HDs)) were assessed using the *Anymaze* computerized tracking system.

##### Open Field

Exploratory behavior and anxiety were assessed in the Open field test using the same apparatus and protocol previously described in Martínez-Cué et al. (2013). During a single 5-min trial, the distance traveled in the center and periphery of the field, and the speed and the number of rearings performed by each animal were assessed with the computerized tracking system *Anymaze*.

##### Exploratory Activity: Hole Board

The apparatus and the protocol employed in this test were identical to those previously described in Martínez-Cué et al. (2013). The number of explorations, the time spent exploring the holes, the number of rearings, and the distance traveled in the apparatus by each mouse over a 5-min period were quantified. To evaluate attention, the repetition or the exploration of recently explored holes (ABA index) was calculated, and this index was corrected by dividing the ABA index by the total number of explorations (ABA/number of explorations).

### β-amyloid Assays

Aβ1-40 and Aβ1-42 levels were assessed in the hippocampi and cortices of the four groups of animals (*n* = 7 per group) by ELISA (kits KMB 3481, and KMB 3441, respectively; Invitrogen, CA, United States) following the protocol described by the manufacturer.

### Determination of TSH and T4

TSH and free T4 were measured in serum obtained from five animals per experimental group by electrochemiluminescence immunoassay (ECLIA) direct sandwich assay, and competitive assay, respectively, automated in an Elecsys e411 (Roche Diagnostics GmbH, Sandhofer Strasse 116, D-68305 Mannheim). Overall intra-assay and inter-assay coefficients of variation were <3% and <7%, and <6% and <10%, respectively. Assay sensitivity was 0.014 microUI/ml and 0.42 ng/dl, respectively.

### Statistics

Shapiro–Wilk tests were used to test the normality of the data sets. Because all the datasets were normally distributed, parametric tests were used. The water maze data from the acquisition sessions (sessions 1–12) was analyzed using two-way Analysis of Variance (ANOVA) with Repeated Measures (RM) (‘session’ x ‘karyotype’ x ‘treatment’ or ‘trial x karyotype x treatment’). The rest of the data was analyzed using two-way (‘karyotype’ x ‘treatment’) ANOVA. The mean values of each experimental group were compared post hoc using Fisher’s LSD (Least Significant Difference) tests. The differences between groups were considered to be statistically significant when *p* < 0.05. All analyses were performed using IBM SPSS Statistics 22 (Armonk, New York, United States) for Windows. .

## Results

### Learning and Memory

#### Morris Water Maze

##### Reference Memory

During the 12 acquisition sessions, all mice reduced their latency to reach the platform across sessions (RM ANOVA ‘session’: *p* < 0.001). TS mice under both treatments exhibited impaired performance with respect to their CO littermates (‘session x karyotype’: *p* = 0.003), and bexarotene treatment diminished the performance of TS but not of CO mice (‘session x treatment’: *p* = 0.016; ‘session x karyotype x treatment’: *p* = 0.041; Figure 1D). When TS and CO mice under both treatments were analyzed separately, it was found that TS mice treated with bexarotene presented diminished performance when compared to their vehicle-treated littermates (‘treatment’: *p* = 0.003), while no significant differences were found between CO mice under bexarotene or vehicle treatment (‘treatment’: *p* = 0.94).

##### Working Memory

RM ANOVA revealed a significant temporal effect when all groups were taken into account (RM ANOVA ‘trial’: *p* = 0.003). However, this effect was due to a reduction in the latency to reach the platform in CO mice, which was not evident in TS mice (‘trial x karyotype’: *p* = 0.001). Bexarotene treatment impaired the working memory of TS mice (‘trial x treatment’: *p* = 0.003; ‘trial x karyotype x treatment’: *p* = 0.050; Figure 1E).

When the four learning curves were analyzed separately, it was found that TS mice under bexarotene (RM ANOVA ‘trial’: *p* = 0.54) or vehicle (*p* = 0.37) did not reduce their latency to reach the platform across trials. Also, TS mice under bexarotene treatment presented higher latencies than TS mice under vehicle treatment (*p* = 0.001). However, both groups of CO mice learned the platform position across trials, as they significantly reduced their latency to reach it within each session (CO Bx: *p* = 0.012; CO Vh: *p* = 0.012; Figure 1E).

##### Cued Sessions

During the cued sessions, no significant differences were found in the latency to reach the platform between both groups of TS and CO mice (‘karyotype’: *p* = 0.11). However, bexarotene treatment increased the latency to reach the visible platform in TS mice (‘treatment’: *p* = 0.23; ‘karyotype x treatment’: *p* = 0.16; Figure 1F).

##### Spatial Memory

TS mice demonstrated diminished spatial memory as demonstrated by the fewer times that they crossed over the place where the platform was located in previous sessions (ANOVA ‘karyotype’: *p* = 0.002; Figure 1G), and entered into the trained quadrant fewer times (‘karyotype’: *p* = 0.043; Figure 1H).

Bexarotene treatment reduced the number of crossings over the platform position (‘treatment’: *p* = 0.032; ‘karyotype x treatment’: *p* = 0.67; Figure 1G), and the number of entries into the trained quadrant (‘treatment’: *p* = 0.038; ‘karyotype x treatment’: *p* = 0.48; Figure 1H) performed by TS mice.

The only group that spent significantly more time in the training quadrant than in the rest of the quadrants was the CO Vh group (ANOVA ‘quadrant’: CO Vh: *p* = 0.010; CO Bx: *p* = 0.40; TS Vh: *p* = 0.53; TS Bx: *p* = 0.83; Figure 1I).

#### Fear Conditioning

No significant differences were found in the time that the four groups of animals spent freezing during the training session (‘karyotype’: *p* = 0.13; Figure 2B). However, TS mice spent less time freezing during the tone-cued test (‘karyotype’: *p* = 0.005) and during the contextual test session (‘karyotype’: *p* = 0.002; Figure 2B). Bexarotene treatment did not exert any effect in the training session (‘treatment’: *p* = 0.45; ‘karyotype x treatment’: *p* = 0.69), or in the tone-cued test session (‘treatment’: *p* = 0.35; ‘karyotype x treatment’: *p* = 0.23), but it increased the time that TS, but not CO mice spent freezing during the contextual testing session (‘treatment’: *p* = 0.17; ‘karyotype x treatment’: *p* = 0.029; Figure 2B).

### Plus Maze

**FIGURE 3:**
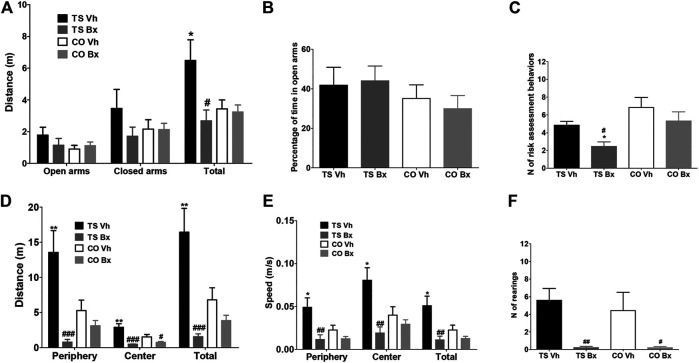
Mean ± S.E.M. of the distance traveled in the open and closed arms, and of the total distance **(A)**, of the percentage of time spent in the open arms **(B)**, and of the number of risk assessment behaviors **(C)**, performed by the four groups of mice in the Plus maze; and of the total distance traveled during the open field test **(D)**, of the speed displayed in the different areas of the apparatus, and the mean speed during the test **(E)**, and of the total number of rearings **(F)**, performed by the four groups of mice. *: *p* < 0.05; **: *p* < 0.01 TS Vh vs. CO Vh, or TS Bx vs. CO Bx; #: *p* < 0.05, ##: *p* < 0.01; ###: *p* < 0.001 TS Bx vs. TS Vh, or CO Bx vs. CO Vh. Fisher’s LSD post-hoc tests.

In the Plus Maze test, vehicle-treated TS mice tended to be hyperactive, since they traveled a longer total distance than the rest of the animals, and bexarotene treatment normalized the locomotor activity of TS mice, although this effect only reached statistical significance when the total distance was taken into account (open arms: ‘karyotype’: *p* = 0.19, ‘treatment’: *p* = 0.53, ‘karyotype x treatment’: *p* = 0.22; closed arms: ‘karyotype’: *p* = 0.52, ‘treatment’: *p* = 0.21, ‘karyotype x treatment’: *p* = 0.22; total distance: ‘karyotype’: *p* = 0.10, ‘treatment’: *p* = 0.018, ‘karyotype x treatment’: *p* = 0.030; Figure 3A).

The four groups of mice did not differ in the percentage of time that they spent in the open arms (‘karyotype’: *p* = 0.18, ‘treatment’: *p* = 0.85, ‘karyotype x treatment’: *p* = 0.63; Figure 3B). Finally, TS mice performed fewer risk assessment behaviors than CO mice (‘karyotype’: *p* = 0.006), and bexarotene treatment reduced the number of these behaviors in TS mice when compared to their vehicle-treated TS littermates (‘treatment’: *p* = 0.027, ‘karyotype x treatment’: *p* = 0.59; Figure 3C).

### Open Field

In the Open Field test, vehicle-treated TS mice presented marked hyperactivity in all zones of the apparatus, and bexarotene treatment reduced the distance traveled by TS and CO mice when compared to vehicle-treated mice of the same karyotype (distance in the periphery: ‘karyotype’: *p* = 0.11, ‘treatment’: *p* < 0.001, ‘karyotype x treatment’: *p* = 0.008; distance in the center: ‘karyotype’: *p* = 0.10, ‘treatment’: *p* < 0.001, ‘karyotype x treatment’: *p* = 0.031; total distance: ‘karyotype’: *p* = 0.09, ‘treatment’: *p* < 0.001, ‘karyotype x treatment’: *p* = 0.006; Figure 3D). In addition, bexarotene-treated TS mice showed marked hypoactivity when compared to vehicle-treated CO mice (Figure 3D).

Vehicle-treated TS mice were faster in all areas of the open field than the rest of the groups of mice, while bexarotene treatment reduced the speed of TS and CO mice with respect to their vehicle-treated littermates of the same karyotype (speed in periphery: ‘karyotype’: *p* = 0.091, ‘treatment’: *p* = 0.002, ‘karyotype x treatment’: *p* = 0.040; speed in center: ‘karyotype’: *p* = 0.18, ‘treatment’: *p* = 0.002, ‘karyotype x treatment’: *p* = 0.037; mean speed: ‘karyotype’: *p* = 0.09, ‘treatment’: *p* = 0.002, ‘karyotype x treatment’: *p* = 0.030; Figure 3E).

TS and CO mice did not differ in the number of rearings performed (‘karyotype’: *p* = 0.67; Figure 3E), while bexarotene treatment reduced the number of rearings performed by mice of both karyotypes (‘treatment’: *p* = 0.002, ‘karyotype x treatment’: *p* = 0.68; Figure 3E).

### Hole Board

**FIGURE 4:**
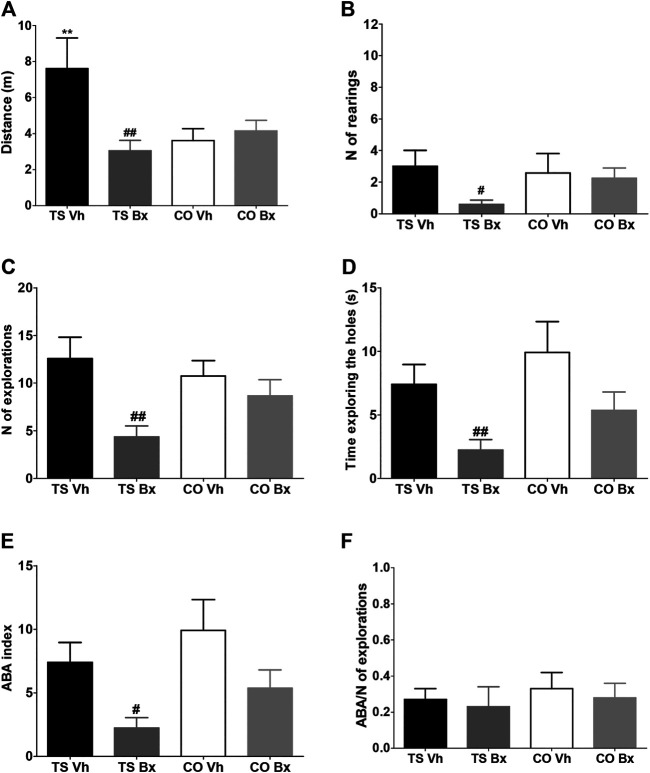
Means ± S.E.M. of the distance traveled **(A)**, the number of rearings **(B)**, the number of explorations **(C)**, the time spent exploring the holes **(D)**, the number of repetitions of previously explored holes (ABA index, E), and the ratio of the number of repetitions of the exploration and the total number of explorations (ABA index/number of explorations **(F)**, performed by TS and CO mice under bexarotene or vehicle treatment in the hole board test. **: *p* < 0.01 TS Vh vs. CO Vh; #: *p* < 0.05, ##: *p* < 0.01 TS Bx vs. TS Vh. Fisher’s LSD post-hoc tests.

Vehicle-treated TS mice traveled a longer total distance than the rest of the groups of animals, bexarotene treatment normalized the activity of TS mice but did not have any effect in CO mice (‘karyotype’: *p* = 0.091, ‘treatment’: *p* = 0.002, ‘karyotype x treatment’: *p* = 0.040; Figure 4A).

Bexarotene treatment also reduced vertical activity (‘karyotype’: *p* = 0.89, ‘treatment’: *p* = 0.25, ‘karyotype x treatment’: *p* = 0.039; Figure 4B), the total number of explorations (‘karyotype’: *p* = 0.10, ‘treatment’: *p* = 0.006, ‘karyotype x treatment’: *p* = 0.85; Figure 4C), and the total exploration time (‘karyotype’: *p* = 0.48, ‘treatment’: *p* = 0.005, ‘karyotype x treatment’: *p* = 0.084; Figure 4D) in TS, but not in CO mice.

Bexarotene treatment reduced the number of repetitions of recently explored holes that TS and CO mice performed (ABA index: ‘karyotype’: *p* = 0.072, ‘treatment’: *p* = 0.006, ‘karyotype x treatment’: *p* = 0.90; Figure 4E). We corrected the ABA index by dividing it by the total number of repetitions and no significant differences were found between the four groups of mice (‘karyotype’: *p* = 0.58, ‘treatment’: *p* = 0.59, ‘karyotype x treatment’: *p* = 0.93; Figure 4F).

### β-amyloid Peptides

**FIGURE 5:**
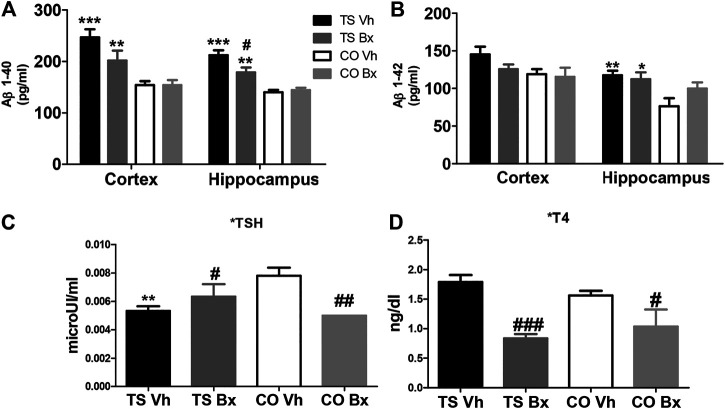
Means ± S.E.M. of the levels of Aβ1-40 **(A)**, and Aβ1-42 **(B)** in the cortices and hippocampi of TS and CO mice under bexarotene or vehicle treatment and of the levels of TSH **(C)**, and T4 **(D)** in the four groups of mice. *: *p* < 0.05, **: *p* < 0.01; ***: *p* < 0.001 TS Vh vs. CO Vh, or TS Bx vs. CO Bx; #: *p* < 0.05; ##: *p* < 0.01; ###: *p* < 0.001 TS Bx vs. TS Vh, or CO Bx vs. CO Vh. Fisher’s LSD post-hoc tests.

TS mice presented higher levels of Aβ1-40 than CO mice in the cortex (‘karyotype’: *p* < 0.001) and in the hippocampus (‘karyotype’: *p* < 0.001; Figure 5A). Bexarotene reduced these levels in the hippocampi of TS, but not CO mice (‘treatment’: *p* = 0.63; ‘karyotype x treatment’: *p* = 0.020). No significant effect was found after bexarotene treatment in Aβ1-40 levels in the cortices of TS or CO mice (‘treatment’: *p* = 0.11; ‘karyotype x treatment’: *p* = 0.11; Figure 5A).

Aβ1-42 levels were higher in TS mice than in CO mice, although this effect only reached statistical significance in the hippocampus (karyotype’: hippocampus: *p* = 0.006; cortex: *p* = 0.053; Figure 5B). Bexarotene treatment did not modify Aβ1-42 levels in the hippocampi (‘treatment’: *p* = 0.31; ‘karyotype x treatment’: *p* = 0.11) or cortices (‘treatment’: *p* = 0.27; ‘karyotype x treatment’: *p* = 0.11; Figure 5B) of TS or CO mice.

### Thyroid Hormones

No significant differences were found in the levels of T4 between TS and CO mice (‘karyotype’: *p* = 0.71). However, bexarotene treatment produced a marked reduction in T4 levels in all animals (‘treatment’: *p* < 0.001, ‘karyotype x treatment’: *p* = 0.83; Figure 5D). In addition, vehicle-treated TS mice showed lower levels of TSH than vehicle-treated CO mice, and bexarotene increased the levels of this hormone in TS mice, while it reduced them in CO mice (‘karyotype’: *p* = 0.64; ‘treatment’: *p* = 0.37, ‘karyotype x treatment’: *p* = 0.002; Figure 5C).

## Discussion

Administration of bexarotene to aged TS mice and their CO littermates for 9 weeks diminished the reference, working, and spatial learning and memory of TS mice, and the spatial memory of CO mice in the MWM. Bexarotene treatment increased the freezing time in the contextual memory test and produced marked hypoactivity in the plus maze, open field, and hole board tests in TS mice, and in the open field and hole board tests in CO mice. Although the administration of bexarotene reduced Aβ1-40 levels in the hippocampi of TS mice, it did not significantly modify these levels in the cortices, or the levels of Aβ1-42 in the cortices or hippocampi of TS or CO mice. Chronic bexarotene administration increased TSH levels in TS mice and reduced TSH levels in CO mice, while animals of both karyotypes displayed reduced T4 levels after bexarotene administration. Finally, chronic bexarotene treatment did not significantly modify the weight of the animals throughout the treatment. The mean weight of both groups of TS mice was lower, while bexarotene-treated CO mice displayed higher body weights.

In the present study, bexarotene administration impaired the cognitive abilities of aged TS mice with AD-like neuropathology in the MWM. In the fear conditioning test, bexarotene increased the freezing time of TS mice in the contextual memory test, which could be indicative of enhanced memory of the association between the context and the aversive stimuli. Previous studies on the effect of bexarotene in different mouse models of AD, including APP/PS1, APP/PS1ΔE9, APP/E3 and APP/E4, APP/PS1-21, Tg2576, Tg, and 3xTg-AD mice concluded that this molecule enhanced the cognitive abilities of these animals in different cognitive tests (i.e. the fear conditioning task, nest building, novel object recognition, the Radial-arm Water Maze task (RWM) and the MWM) (Cramer et al., 2012; Fitz et al., 2013; Landreth et al., 2013; Tesseur et al., 2013; Muñoz-Cabrera et al., 2020).

Cramer et al. (2012) proposed that the cognitive improvements exerted by bexarotene were due to the ApoE–dependent clearance of soluble Aβ peptides from the brain and the reduction of amyloid plaques. However, none of the aforementioned studies (Fitz et al., 2013; Landreth et al., 2013; Tesseur et al., 2013; Boehm-Cagan and Michaelson, 2014) were able to replicate this reduction in plaque burden. There is also controversy regarding the ability of bexarotene to reduce the Aβ load in the brains of these animals. While some studies found no reduction of Aβ levels after bexarotene treatment (Price et al., 2013; Tesseur et al., 2013), others (Fitz et al., 2013; Veeraraghavalu et al., 2013) found reductions similar to the ones reported by Cramer et al. (2012), indicative of the ability of this compound to enhance the clearance of these peptides. In the present study, we found a reduction of Aβ1-40 in the hippocampi of TS animals and a similar tendency in their cortices which did not reach statistical significance. No changes were induced by bexarotene in the levels of Aβ1-42 in the cortices or hippocampi of CO mice. Consistent with these results, O’Hare et al. (2016) did not find changes in any of these peptides in the cortices of a mouse model of AD. Furthermore, Veeraghavalu et al. (2013) reported a reduction of soluble Aβ1-40, but not of Aβ1-42 expression in the brains of APP/PS1 mice. It is possible that the different models used and differences in the methods of assessing Aβ load, the doses, the duration of the treatments, and the ages of the animals are responsible for these discrepancies.

Different mechanisms might also be responsible for the changes in the Aβ burden in those studies where it was evident. All of them reported that bexarotene acted on astrocytes inducing the expression of ApoE and ABCA1. Landreth et al. (2013) proposed that because the induction of these genes increases the production of ApoE-containing high-density lipoprotein (HDL) particles, which causes the proteolytic degradation of Aβ peptides, this thereby facilitated their clearance. These effects could be partially responsible for the cognitive benefits found after bexarotene administration in some studies.

Another putative mechanism for the bexarotene-induced cognitive improvement could be its beneficial effects on synaptic integrity. Tachibana et al. (2016) found that several key synaptic proteins that regulate plasticity (i.e., PSD95, Glutamate Receptor 1 (GluR1), and N-methyl-d-aspartate Receptor NR1 subunit (NR1)), which are reduced in aged animals, are restored in the brains of wild-type mice after bexarotene treatment. In addition, Muñoz-Cabrera et al. (2020) reported a recovery of basal synaptic transmission and synaptic plasticity in 3xTg-AD mice after bexarotene administration.

Neuroinflammation has been demonstrated to be both an inducer and a result of amyloid pathology in AD (Martínez-Cué and Rueda, 2020). Also, TS mice have an enhanced neuroinflammatory response; thus, changes in the levels of pro-inflammatory cytokines could play a role in the AD phenotypes present in these animals. Bexarotene has been demonstrated to reduce numerous pro-inflammatory cytokines (IL-1β, IL-2, IL-6, IL-10, IL-12, IFN-γ, TNF-α, G-CSF, GM-CSF), while not affecting the levels of IL-17 A or IL1A in mice (Janakiram et al., 2012). A recent study demonstrated that TS mice present increased levels of IL-1β, IFN-γ, G-CSF, and IL-17A. The administration of an antibody against IL17-A normalized the levels of these cytokines, improved their cognitive abilities, reduced cellular senescence, and normalized Aβ-42 levels in the hippocampi of aged TS mice, but did not affect Aβ1-40 levels in the cortices or hippocampi of these animals (Rueda et al., 2018). Because of its ability to induce on its own or to work synergistically with IL1β and IFNγ to induce the expression of other pro-inflammatory cytokines (Korn et al., 2009; Meares et al., 2012; Zimmermann et al., 2018), IL-17A plays a prominent role in the induction of the neuroinflammation implicated in the onset of different hallmarks of AD, including the increase in Aβ load. Thus, the inability of bexarotene to reduce the expression of IL-17A may be partially responsible for its lack of effects on Aβ1-42 levels and/or to its failure to improve the cognitive abilities of TS mice found in the present study.

Several studies have also failed to find positive effects of bexarotene administration on the cognitive abilities of different models of AD. O’Hare et al. (2016) did not find any difference in the MWM between bexarotene- and vehicle-treated APPSwFILon, PSEN1*M146L*L286V, or between bexarotene- or vehicle-treated rats after the administration of Aβ species. Also, in these animals bexarotene did not exert any effect on their long-term potentiation. These results are partially consistent with our results. As mentioned above, in this study, chronic bexarotene treatment diminished the reference, working, and spatial learning and memory in aged TS mice, while it did not exert any effect on reference or working memory in CO mice and only diminished their spatial memory in the probe trial in the MWM. In agreement with these results, various different studies have failed to find any positive effect of bexarotene in different cognitive tasks performed in normal rodents such as the odor recognition test in C57Bl6 mice (Cramer et al., 2012), or the MWM for either B6SJ mice (O’Hare et al., 2016), or aged CD1 mice (Monroy et al., 2020). A putative explanation of the inconsistencies between these results and the ones reporting pro-cognitive effects of bexarotene was proposed by Tesseur et al. (2013). These authors stated that some side-effects of bexarotene may confound the interpretation of the cognitive tests. In their study, social recognition memory was improved after bexarotene treatment but in the retention test of the passive avoidance task, bexarotene-treated hAPP/PS1 mice showed longer step-through latency. This effect could be due to the reduced exploratory activity that they found in hAPP/PS1 mice after bexarotene treatment. Similarly, in the present study, we observed a marked reduction in the activity of bexarotene-treated TS mice in the open field, hole board, and plus maze tests. This hypoactivity might be responsible for the increased latency to reach the platform in the MWM during the acquisition sessions, and the increased freezing time in the fear condition test displayed by TS mice after bexarotene administration. Bexarotene-treated aged CO mice only presented reduced activity in the hole board and in the open field tests, which might be indicative of a slighter hypoactivity effect that could explain why their performance was not affected during the acquisition sessions in the MWM or the fear conditioning test. Monroy et al. (2020) did not find any effect of bexarotene on the locomotor activity of aged CD1 mice in a novel environment. Altogether, this might indicate that hypoactivity in normal rodents might be less marked and/or only detected in specific experimental conditions.

The percentage of time spent in each quadrant during the probe trial is a measure of spatial memory which is not dependent on the animals’ level of activity. The present study demonstrated a reduction of this type of memory in both TS and CO mice. Because PPARγ, LXR, and RXR receptors regulate ApoE expression by forming heterodimers with each other or with other nuclear receptors including Thyroid hormone receptor (TR) (Chawla et al., 2001; Szanto et al., 2001; Sussman and de Lera, 2005; Evans and Mangelsdorf, 2014), the modulation of these receptors induced by bexarotene might have induced alterations in thyroid regulation in those mice treated with bexarotene. Indeed, according to the Federal Drug Administration’s (FDA) approval status (Lowednthal et al., 2012), bexarotene can induce hypothyroidism. Thus, bexarotene-induced thyroid disorders may represent a putative mechanism for the cognitive deterioration observed in these animals after its chronic administration.

Individuals with DS have a higher incidence of thyroid dysfunction, including subclinical hypothyroidism, congenital hypothyroidism, and thyroid autoimmunities (such as Hashimoto’s disease or Grave’s disease) than the normal population (Whooten et al., 2018). Up to 24% of individuals with DS have subclinical hypothyroidism and are more likely to progress to overt hypothyroidism (Whooten et al., 2018). In the present study, although TS and CO mice under vehicle treatment showed similar levels of T4, TS animals presented lower levels of TSH than CO mice, which might indicate that under basal conditions, trisomic mice have subjacent thyroid alterations but not hypothyroidism. However, bexarotene increased the levels of TSH and reduced the levels of T4 in TS mice when compared to their vehicle-treated TS littermates, indicating that bexarotene was inducing primary hypothyroidism in these animals. In contrast to this data, the vast majority of studies in the literature had associated chronic administration of bexarotene with central hypothyroidism (Makita et al., 2019). In this regard, in the case of CO mice, bexarotene reduced the levels of both hormones, which suggests that these animals might be suffering from secondary hypothyroidism. Taken together, this suggests that thyroid disorders related to bexarotene administration may have different pathogenic mechanisms. Thus, differences in thyroid function might be partially responsible for the higher deterioration of cognition and behavior found in TS when compared with CO mice. Because hypothyroidism is associated with fatigue and attentional and memory dysfunctions, this side effect of bexarotene is a likely explanation for the diminished performance in the behavioral and cognitive tests found in the treated animals in this study.

Finally, another common symptom of hypothyroidism is weight gain, which could also have compromised the performance of the experimental animals. In rodent studies, bexarotene has been associated with weight loss (Tesseur et al., 2013; Tachibana et al., 2016), weight gain (Riancho et al., 2015), or lack of change in body weight (Fitz et al., 2013). In the present work, bexarotene did not significantly modify the bodyweight of TS or CO mice (data not shown). The different animal models used, their ages, the doses of bexarotene as well as the duration of its administration could be responsible for these discrepancies.

In conclusion, the administration of bexarotene diminished the performance of TS and CO mice in the MWM and produced a marked hypoactivity in the rodents. These effects are likely to be due to the induction of hypothyroidism, demonstrated by the reduced levels of T4 in TS and CO mice. Although the administration of bexarotene reduced Aβ1-40 levels in the hippocampi of TS mice, it did not significantly modify these levels in their cortices, or the levels of Aβ1-42 in the cortices or hippocampi of TS or CO mice. These results do not provide support for the use of bexarotene as a potential treatment of AD neuropathology in the DS population.

## Data Availability

The raw data supporting the conclusions of this article will be made available by the authors, without undue reservation.

## Glossary

Aβ: β- amyloid;
AD: alzheimer’s disease
ALS: amyotrophic lateral sclerosis
ANOVA: analysis of variance
ApoE: apolipoprotein E
Bx: bexarotene
CO: control
CO Bx: control bexarotene
CO Vh: control vehicle
DS: down syndrome
ECLIA: electrochemiluminescence immunoassay
FDA: federal drug administration
GluR1: glutamate receptor 1
HD: head dippings
HDL: high-density lipoprotein
LSD: least significant difference
LXR: liver X receptor
MWM: morris water maze
NFT: neurofibrillary tangles
NR1: N-methyl-D-aspartate receptor subunit 1 (NR1)
PPARγ: Peroxisome Proliferator‐Activated Receptor‐c
RAR: retinoid acid receptor
RM: repeated measures
RXR: retinoid X receptor
SAP: stretch-attend postures
T4: thyroxine
TR: thyroid hormone receptor
TS: Ts65Dn
TS Bx: Ts65Dn bexarotene
TSH: Thyroid-stimulating hormone
TS Vh: Ts65Dn vehicle
VDR: vitamin D receptor
Vh: vehicle
